# *Pueraria lobata* Extract Protects Hydrogen Peroxide-Induced Human Retinal Pigment Epithelial Cells Death and Membrane Permeability

**DOI:** 10.1155/2019/5710289

**Published:** 2019-08-27

**Authors:** Nu Ri Kang, Bo-Jeong Pyun, Dong Ho Jung, Ik Soo Lee, Chan-Sik Kim, Young Sook Kim, Jin Sook Kim

**Affiliations:** ^1^Herbal Medicine Division, Korea Institute of Oriental Medicine (KIOM), Daejeon, Republic of Korea; ^2^Korean Medicine Life Science, University of Science Technology (UST), 217 Gajeong-ro, Yuseong-gu, Daejeon, Republic of Korea; ^3^Drug Development Team, PharmAbcine, Yuseong-gu, Daejeon, Republic of Korea; ^4^Research Infrastructure Team, Korea Institute of Oriental Medicine (KIOM), Daejeon, Republic of Korea; ^5^Clinical Medicine Division, Korea Institute of Oriental Medicine (KIOM), Daejeon, Republic of Korea

## Abstract

**Background:**

Pueraria *lobata* is used in traditional Asian medicine to treat cardiovascular diseases, diarrhea, diabetes mellitus, and diabetic complications such as diabetic retinopathy. Oxidative stress in retinal pigment epithelial cells is implicated in the pathogenesis of retinopathy and age-related macular degeneration (AMD). Here, we evaluated whether the *P. lobata* extract can prevent cell death and decrease membrane permeability in oxidative stress-induced human retinal pigment epithelial cells.

**Methods:**

The effects of *P. lobata* extract on hydrogen peroxide- (H_2_O_2_-) induced oxidative stress were investigated using 2′,7′–dichlorofluorescin diacetate, western blotting, and immunohistochemistry in human retinal pigment epithelial cells. The effects of puerarin, daidzein, and daidzin isolated from *P. lobata* extract were also studied by determining cell death, reactive oxygen species (ROS) generation, and p38 mitogen-activated protein kinase (MAPK) and c-Jun *N*-terminal kinase (JNK) phosphorylation.

**Results:**

Our results showed that the *P. lobata* extract inhibited ROS generation, suppressed the disruption of zonula occludens-1 (ZO-1), and reduced membrane permeability in H_2_O_2_-induced human retinal pigment epithelial cells. Additionally, the *P. lobata* extract prevented the inhibition of p38 MAPK and JNK phosphorylation.

**Conclusion:**

Our findings suggest that the *P. lobata* extract has the potential to prevent AMD development by inhibiting the mechanism underlying oxidative stress-mediated ocular disorders.

## 1. Background

Retinal damage, also known as retinopathy, is a major cause of vision loss among middle-aged and elderly people, often resulting as a consequence of complications associated with diabetes, hypertension, atherosclerosis, blood dyscrasias, systemic infections, and exposure to radiation [1–3]. The retinal pigment epithelium (RPE) plays an important role in the development and maintenance of adjacent photoreceptors in the retina. RPE cells are used to investigate the pathology and physiology of diabetic retinopathy and age-related macular degeneration (AMD). Drug candidates are tested in RPE cells to develop treatments for retinopathy and AMD [4].

Oxidative stress plays a pivotal role in the development and acceleration of retinal diseases. It increases intracellular levels of reactive oxygen species (ROS), which cause retinal damage, and is a major pathogenic component [5]. ROS increase the chronological age of cells and reduce mitochondrial function in RPE cells, causing cell damage [6]. A recent study showed that oxidative stress has a particularly significant role in the development, degeneration, dysfunction, and age-related loss of RPE [7, 8]. Repeated exposure to oxidative stress from ROS, such as hydrogen peroxide (H_2_O_2_), causes RPE damage [9]. Therefore, H_2_O_2_ is suitable for evaluating oxidative damage of RPE and investigating retinopathy progression. In addition, oxidative stress influences the formation of the blood-retinal barrier (BRB) by RPE via tight junctions and adherens junctions [9–11]. The tight junction consists of the transmembrane protein zonula occludens-1 (ZO-1) and occludins, which maintain BRB integrity [9, 10]. Oxidative stress disrupts the tight junctions and increases paracellular permeability across the epithelial monolayers, thereby decreasing the localization of occludins and association of ZO-1 [10, 12].

The roots of *Pueraria lobata* Ohwi (family: Fabaceae) are well known and used in traditional medicine. The plants are widely cultivated in East Asia and used for the treatment of diarrhea, diabetes, and cardiovascular diseases [13–15]. *P. lobata* extract and the compounds present in the extract have been shown to possess therapeutic properties, owing to their antioxidant, anti-ischemic, anticancer, anti-inflammation, antifatigue, and antiretinopathic effects [14–17]. A previous study showed that the *P. lobata* extract prevent apoptosis of lung fibroblasts in Chinese hamsters by inhibiting hydrogen peroxide-induced oxidative stress [18]. In this study, we explored whether the *P. lobata* extract and their individual constituent compounds (puerarin, daidzein, and daidzin) can protect human RPE cells against oxidative stress. In addition, we evaluated the expression of tight junctions and oxidative stress-induced decrease in cell membrane permeability as well as examined the mechanisms involved in the antioxidative effects of *P. lobata* in RPE cells.

## 2. Methods

### 2.1. Extraction of *P. lobata* and Isolation of Single Compounds

**Figure 1:**
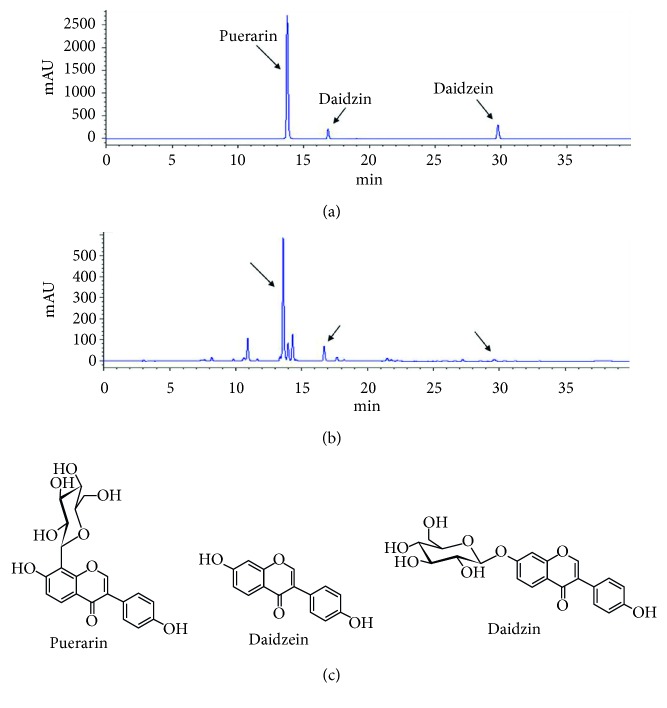
HPLC chromatographs of the *P. lobata* extract and chemical structures of single compounds. Standard mixture (a) and *P. lobata* extract (b) with detection at 254 nm. Chemical structures of puerarin, daidzein, and daidzin isolated from *P. lobata* (c).

The roots of *P. lobata* Ohwi (*P. lobata*) were collected from Kyonggi-do, Gachun University, Korea, and were identified by Professor J.-H. Kim. A voucher specimen (no. KIOM-P041) has been deposited at the Herbarium of Korea Institute of Oriental Medicine. The air-dried plant material (4.9 kg) was extracted with 20 L of EtOH three times by maceration. The extract was combined and concentrated *in vacuo* at 40°C to yield an EtOH extract (665 g). This extract was subjected to a series of chromatographic procedures, using open silica gel and RP-18 column and HPLC, leading to the isolation of three main compounds. By comparing their physicochemical and spectral data with those in the literature [19], these compounds were identified as puerarin, daidzein, and daidzin (Figure 1).

### 2.2. Cell Culture

Human RPE cells (ARPE-19) were purchased from ATCC (Manassas, VA) and maintained in Ham's F-12 : Dulbecco's Modified Eagle's Medium (1 : 1) containing 10% fetal bovine serum (FBS, Gibco, USA). The cells were cultured at 37°C under 5% CO_2_ in a humidified incubator, and the culture medium was replaced every 48 h. After the seeded cells reached confluency, they were rinsed with phosphate-buffered saline (PBS) and then incubated with 0.5% trypsin-EDTA (Gibco) for 5 min at 37°C.

### 2.3. Cell Viability Assay

Cell viability was determined using the cell counting kit-8 (CCK-8) assay (Dojindo Molecular Technologies, Japan). RPE cells (0.5 × 10^4^ cells/well) were seeded into each well of a 96-well plate containing F12/DMEM with 10% FBS and then were incubated for 24 h. After cell attachment, the cells were pretreated with the *P. lobata* extract and its individual compounds (puerarin, daidzein, and daidzin) in serum-free medium for 1 h and with H_2_O_2_ for 24 h. After incubation, 10 *μ*L of CCK-8 was added to each well of the 96-well plate and incubated at 37°C under 5% CO_2_ in a humidified incubator for 2 h. The absorbance was measured at 450 nm using a Multidetection Microplate Reader (BioTek, Synergy HT, Winooski, VT).

### 2.4. Measurement of ROS Production

ROS production was measured by using the 2′,7′–dihydrodichlorofluorescein diacetate (DCF-DA, Invitrogen, USA) staining assay. After pretreatment with the *P. lobata* extract, puerarin, daidzein, and daidzin for 1 h, the cells were cotreated with H_2_O_2_ (200 *μ*M) and 25 *μ*M DCF-DA in a humidified 5% CO_2_ incubator at 37°C for 30 min. The cells were then washed with PBS and treated with 0.5% trypsin-EDTA. After the cells were detached, they were harvested in PBS. ROS production was immediately analyzed by using BD FACSCalibur™ (San Jose, CA, USA).

### 2.5. Permeability Assay

To evaluate the inhibitory effect of the *P. lobata* extract on oxidative stress-induced alteration in cell membrane permeability, the cells were seeded on a Transwell upper chamber (24 wells, PE, 0.4 *μ*m pore diameter, Corning Inc., Tewksbury, MA, USA) at a density of 0.3 × 10^4^ cells/well. After the cells formed a monolayer, the medium was replaced with a serum-free medium. The *P. lobata* extract, puerarin, daidzein, and daidzin as well as FITC-conjugated dextran (50 kDa; Sigma-Aldrich, St. Louis, MO, USA) were added into the Transwell and incubated for 1 h. Next, H_2_O_2_ (200 *μ*M) was added and the plate was incubated for 24 h at 37°C under 5% CO_2_ in a humidified incubator. FITC was measured at an excitation wavelength of 485 nm and an emission wavelength of 525 nm using Synergy™ HT Multidetection Microplate Reader (BioTek).

### 2.6. Immunohistochemistry

After treatment, the cells were washed with PBS and fixed with 2% paraformaldehyde for 20 min at 4°C. Next, the cells were washed twice with PBS and permeabilized using 0.2% Triton X-100 in PBS for 15 min at room temperature. Then, they were again washed twice and blocked using blocking buffer (PBS containing 0.3% Triton X-100 and 5% normal serum) at room temperature. The cells were subsequently incubated with a primary antibody against ZO-1 in blocking buffer (1 : 1000) overnight at 4°C, washed three times with PBS, and incubated with a secondary antibody in blocking buffer (1 : 1000) for 2 h at room temperature. The cells were then treated with DAPI for visualization of the nuclei. Immunofluorescent images of the cells were captured using a fluorescence microscope (BX51, Olympus microscope, Japan).

### 2.7. Western Blot Analysis

After treatment, the cells were washed with PBS and harvested using Laemmli sample buffer (Bio-Rad, Hercules, CA, USA). Total protein concentrations were determined using a BCA Protein Assay Kit (Pierce Chemical, Grand Island, NY, USA). To prepare the protein samples for analysis, they were boiled. The sample was separated into equal amounts by 10% SDS-PAGE gel electrophoresis and transferred to a nitrocellulose blotting membrane (GE Healthcare Life Science, Germany). The membranes were washed with Tris-buffered saline containing 0.1% Tween-20 (TBST) and then incubated with a diluted primary antibody in TBST overnight at 4°C. The membranes were then washed three times with TBST and incubated with a diluted secondary antibody in TBST for 2 h at room temperature. The membranes were further washed with TBST and detected using EzWestLumi One (ATTO Corp., Japan) with LAS 3000 (Fujifilm, Tokyo, Japan).

### 2.8. Statistical Analysis

The data are expressed as the mean ± SEM of multiple experiments. Paired Student's *t*-tests were used to compare two groups, and ANOVA with Tukey's test was used for multiple comparison tests using Prism 5.0 software (GraphPad 5.0, San Diego, CA, USA). Values of *p* < 0.05 indicated statistical significance.

## 3. Results

### 3.1. HPLC Analysis of Puerarin, Daidzein, and Daidzin in *P. lobata* Extract

The HPLC method was applied to the quantitative analysis of puerarin, daidzein, and daidzin in the *P. lobata* extract. The identification and quantitative determination of puerarin, daidzein, and daidzin in the extract were accomplished by a comparison of the retention time and area with those of standard three compounds (Figure 1(b)). The linearity of the HPLC method was checked by injecting five concentrations of standard solutions (Figure 1(a)). The calibration curves of puerarin, daidzein, and daidzin showed good linearity (*r*^2^ > 0.9999) within given concentration ranges (Table 1). The contents of puerarin, daidzein, and daidzin in the *P. lobata* extract were 188.90, 2.73, and 32.54 mg/g, respectively (Table 2).

### 3.2. Effect of *P. lobata* Extract on H_2_O_2_-Induced RPE Cell Death

**Figure 2:**
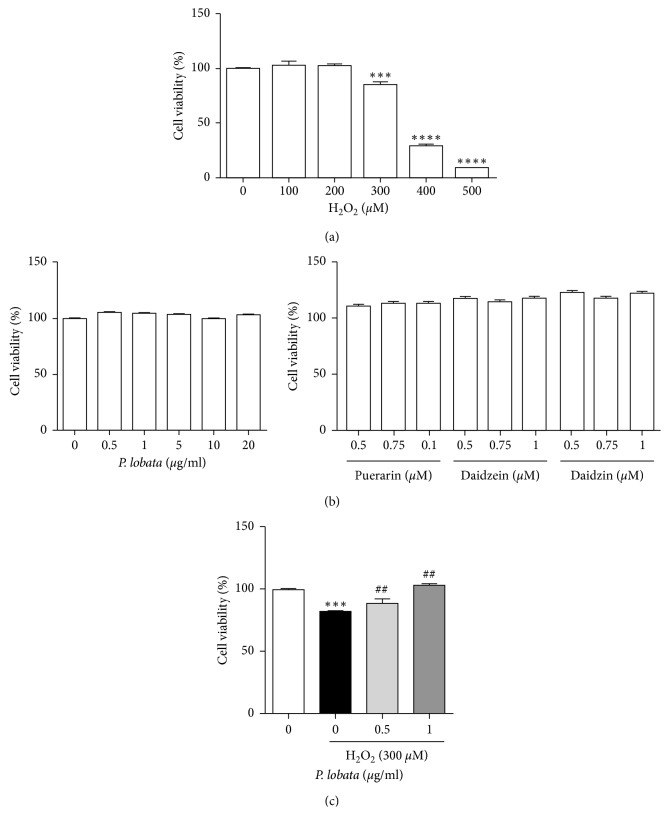
(a) H_2_O_2_-induced RPE cell death. Data are representative of three independent experiments and are expressed as the mean ± SEM. (*n* = 5). ^*∗∗∗*^*p* < 0.001; ^*∗∗∗∗*^*p* < 0.0001 vs. control, respectively. (b) Effect of *P. lobata* extract and its single compounds on cell viability. (c) Inhibitory effect of *P. lobata* extract on H_2_O_2_-induced RPE cell death. Data are representative of three independent experiments and are expressed as the mean ± SEM. (*n* = 6–8). ^*∗∗∗*^*p* < 0.01 vs. control; ^##^*p* < 0.01 vs. H_2_O_2_-induced cell viability.

To examine the concentration of H_2_O_2_ that induced RPE cell death, the CCK-8 assay was performed, which showed that H_2_O_2_ reduced RPE cell viability in a concentration-dependent manner (Figure 2(a)). H_2_O_2_ at 300–500 *μ*M reduced the cell viability index (%) at 24 h in a dose-dependent manner, and the difference was significant compared with the value of the control group (^*∗∗∗*^*p* < 0.001, ^*∗∗∗∗*^*p* < 0.0001 vs. control). Next, to test the effect of the *P. lobata* extract on cell viability, the cells were treated with various concentrations (0.5–20 *μ*g/mL) of the extract for 24 h. A high dose of the extract and single compounds did not alter cell viability (Figure 2(b)). To examine whether the *P. lobata* extract can protect against H_2_O_2_ (300 *μ*M) induced cell death, the cells were treated with the *P. lobata* extract and H_2_O_2_. As shown in Figure 2(c), the *P. lobata* extract (0.5 and 1 *μ*g/mL) significantly inhibited H_2_O_2_-induced cell death (^*∗∗∗*^*p* < 0.001 vs. control; ^#^*p* < 0.05 vs. H_2_O_2_-treated cells).

### 3.3. Effect of *P. lobata* Extract on H_2_O_2_-Induced Intracellular ROS Generation

**Figure 3:**
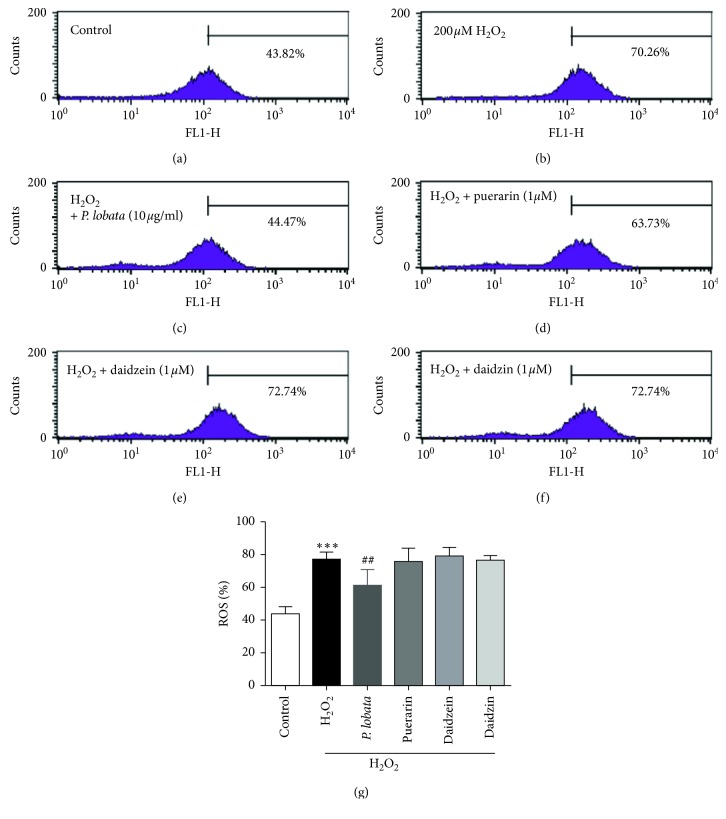
Inhibitory effects of *P. lobata* extract on ROS generation. Intracellular ROS, detected as H_2_DCF-DA fluorescence, was measured using FACS. Cells were pretreated with *P. lobata* extract (10 *μ*g/mL) and its single compounds (1 *μ*M) for 60 min and then cultured for 10 min in the presence of H_2_O_2_ (200 *μ*M). Data are expressed as the mean ± SEM. (*n* = 4). ^*∗∗∗*^*p* < 0.001 vs. control; ^##^*p* < 0.05 vs. H_2_O_2_-induced cells.

To determine the concentration of the *P. lobata* extract and its single compounds that do not affect cell viability, the cells were treated with the *P. lobata* extract and its single compounds and with 200 *μ*M H_2_O_2_ for 24 h. Before evaluating H_2_O_2_-induced intracellular ROS generation, the time required for intracellular ROS generation detected as H_2_DCF-DA was determined by FACS analysis. As expected, intracellular ROS generation was markedly increased for a 30 min treatment with H_2_O_2_ treatment (data not shown). H_2_O_2_ at a concentration of 200 *μ*M showed no effect on cell viability; however, it markedly increased ROS generation (Figures 2(a) and 3(b)). Next, we examined the effect of the *P. lobata* extract and its individual components (puerarin, daidzein, and daidzin) on ROS generation. The *P. lobata* extract (10 *μ*g/mL) inhibited H_2_O_2_-induced intracellular ROS generation, with the levels reaching those of the normal controls (Figures 3(c) and 3(g)). Puerarin, daidzein, and daidzin (1 *μ*M) did not show any effect on H_2_O_2_-induced intracellular ROS generation (Figures 3(d)∼3(f)). Taken together, these data suggest that the *P. lobata* extract could inhibit oxidative damage in RPE cells.

### 3.4. Effect of *P. lobata* Extract on Paracellular Permeability

**Figure 4:**
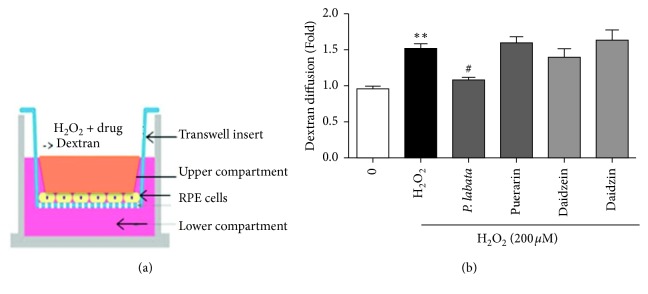
Inhibitory effects of *P. lobata* extract and its single compounds on paracellular permeability. (a) Systems to detect permeability of RPE cells using Transwell insert. (b) After the treatment, FITC-dextran permeability was examined for 90 min. Data indicate that *P. lobata* (10 *μ*g/mL) and its single compounds (1 *μ*M) have an inhibitory effect on H_2_O_2_-induced paracellular permeability in RPE cells. Data are expressed as the mean ± SEM. (*n* = 4). ^*∗∗*^*p* < 0.01 vs. control; ^#^*p* < 0.05 vs. H_2_O_2_-induced cells.

The RPE cells form the outer layer of the BRB, and the tight junctions expressed in the outer BRB regulate entry of fluids and solutes into the retina that are essential for retinal homeostasis. We explored the effect of the *P. lobata* extract on the function of the RPE barrier by measuring paracellular permeability of 50 kDa dextran in RPE cells. As shown in Figure 4(a), cell systems treated with FITC-dextran have been used for in vitro permeability assays [2, 20]. As shown in Figure 4(b), oxidative damage increased the diffusion of FITC-dextran and the *P. lobata* extract inhibited this increase by almost 33.3%. However, the individual compounds (at 1 *μ*M concentration) could not inhibit this oxidative stress-induced diffusion. These results suggest that the *P. lobata* extract could decrease the membrane permeability in oxidative stress-induced RPE cells and protect the BRB.

**Figure 5:**
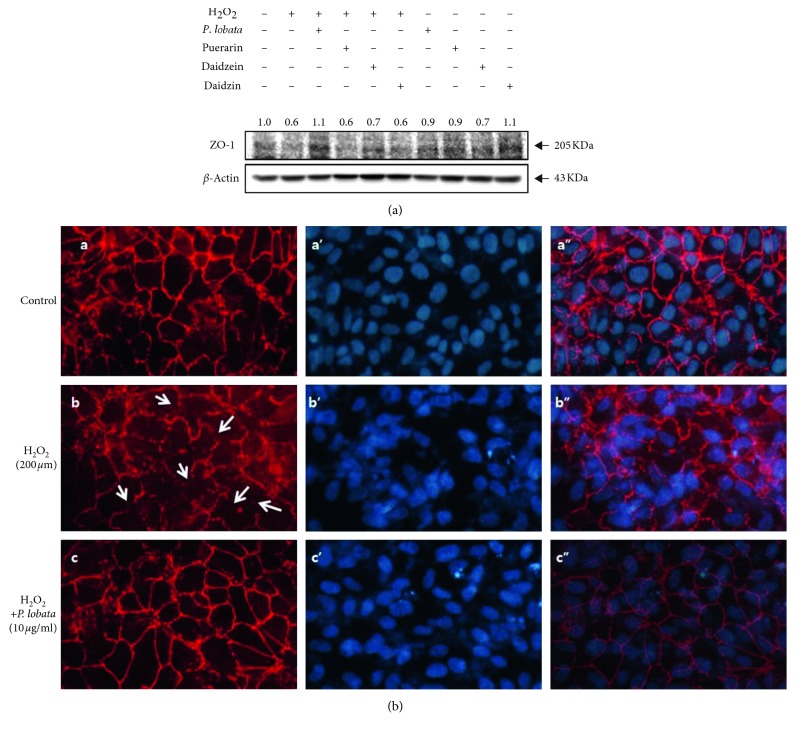
Effect of *P. lobata* on ZO-1 expression in RPE cells. Cells were pretreated with *P. lobata* extract (10 *μ*g/mL) and its single compounds (1 *μ*M) for 60 min and then cultured for 24 h in the presence of H_2_O_2_. (a) Western blotting analysis of ZO-1 expression. (b) Immunohistochemistry for ZO-1 in H_2_O_2-_induced cells treated with *P. lobata* extract. Immunofluorescence of RPE monolayers shows the beneficial effect of *P. lobata* (10 *μ*g/mL) and its single compounds (1 *μ*M) in RPE cells against H_2_O_2_-induced tight junction expression. ZO-1 (a–c, red), DAPI (a'–c', blue), and merged images (a”–c”).

Paracellular permeability is related to the altered expression of tight junction proteins such as ZO-1. We evaluated the alterations in ZO-1 expression in response to oxidative damage for 24 h using immunoblotting. *P. lobata* extract and single compounds were examined for their inhibitory effects. The expression of ZO-1 decreased in RPE cells exposed to oxidative stress, and *P. lobata* extract attenuated the decrease in the expression of ZO-1 as compared to the level of normal control (Figure 5(a)). In addition, ZO-1 expression on cell membranes was evaluated using immunohistochemistry in RPE cells; ZO-1 expression was altered in response to oxidative damage. Fluorescence intensity and areas of discontinuity of ZO-1 were reduced in H_2_O_2_-treated RPE cells compared to those of the normal control (Figure 5(b)-b, white arrows). When treated with *P. lobata* extract, H_2_O_2_-treated RPE cells showed normalization of ZO-1 immunostaining (Figure 5(b)-c). These data demonstrate that the *P. lobata* extract inhibited the disruption of the tight junction protein in oxidative stress-induced RPE cells.

### 3.5. Inhibitory Effects of *P. lobata* Extract on H_2_O_2_-Induced Phosphorylation of p38 MAPK and JNK in RPE Cells

**Figure 6:**
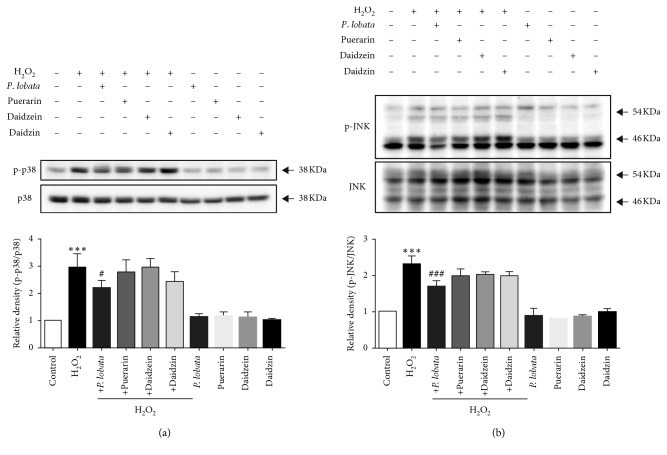
Inhibitory effects of *P. lobata* extract on H_2_O_2_-induced phosphorylation of p38 MAPK and JNK in RPE cells. RPE cells were pretreated with *P. lobata* extract (10 *μ*g/mL) and its single compounds (1 *μ*M) for 30 min, followed by treatment with H_2_O_2_ (200 *μ*M) for 30 min. Phosphorylation of p38 (a) and JNK (b) was detected by western blot analysis. Data are expressed as the mean ± SEM. (*n* = 4). ^*∗∗∗*^*p* < 0.001 vs. control; ^#^*p* < 0.05 and ^###^*p* < 0.001 vs. H_2_O_2_-induced cells, respectively.

To identify the signaling pathway through which the *P. lobata* extract exhibits their effects in H_2_O_2_-treated RPE cells, we examined the effects of the *P. lobata* extract on the phosphorylation of p38 MAPK and JNK.  Figure 6 shows the representative immunoblots of phosphorylated p38 MAPK and JNK in H_2_O_2_-treated RPE cells. Phosphorylation of p38 MAPK and JNK was increased by 3-fold and 2.5-fold, respectively, after H_2_O_2_ treatment in RPE cells. The *P. lobata* extract significantly inhibited phosphorylation of p38 MAPK (Figure 6(a)) and JNK (Figure 6(b)) in RPE cells.

## 4. Discussion

In this study, the *P. lobata* extract were tested for their potential inhibitory effect against H_2_O_2_-induced RPE cell death and membrane permeability. The *P. lobata* extract significantly inhibited cell death and ROS generation. Membrane permeability was prevented, and the expression of the tight junction protein ZO-1 was increased in H_2_O_2_-treated RPE cells following treatment with the *P. lobata* extract. Additionally, H_2_O_2_-induced p38 MAPK and JNK phosphorylation was reduced after treatment of RPE cells with the *P. lobata* extract.

AMD is the leading cause of blindness in the elderly. The pathogenesis of AMD is related to oxidative stress, formation of drusen, accumulation of lipofuscin, and inflammation in the retina. The retina consumes oxygen causing it to be particularly susceptible to oxidative stress. Oxidative stress causes RPE cell death in AMD and then shows a lack of chromatin condensation and DNA fragmentation [21]. One of the targets of many drugs aiming to treat or prevent AMD is the inhibition of oxidative stress or its downstream pathways: inflammation, pathological neovascularization, and miRNA [22–24]. Recently, small-molecule drugs from single compounds were shown to have therapeutic effects in AMD by inhibiting oxidative stress [23]. Curcumin exhibits a strong antioxidant activity, upregulates heme oxygenase-1 (HO-1) (the oxidative stress defense enzyme), and may protect RPE cells against oxidative stress by reducing ROS levels [25]. Paeoniflorin also protects RPE cells against oxidant stress, and canolol prevents oxidative stress-induced cell damage [23, 26].

A previous study showed that the antioxidative activity of *P. lobata* was attributable to higher contents of the isoflavonoids puerarin, daidzein, and daidzin. *P. lobata* water extract and puerarin (IC_50_ value of 756.2 *μ*M) have antioxidant effects against free-radical-mediated damage of red blood cells [27]. Daidzein and daidzin do not possess strong antioxidative effects, with IC_50_ values over 1000 *μ*M [27]. Our results also showed that the *P. lobata* extract had an antioxidant effect in H_2_O_2_-treated RPE cells (Figure 3(g), 10 *μ*g/mL) and prevented cell death (Figure 2(c), 0.5 and 1 *μ*g/mL). However, the individual components (1 *μ*M) from the *P. lobata* extract could not significantly inhibit H_2_O_2_-induced oxidation in RPE cells. These results indicate that the *P. lobata* extract, comprising the three individual components, has synergistic effects on antioxidation in RPE cells. Pretreatment with the *P. lobata* extract showed a preventive effect against H_2_O_2_-induced oxidation and cell death.

Tight junctions primarily establish a permeable retinal barrier across epithelial sheets. Occludin is the first transmembrane protein of tight junctions to be identified, and ZO-1 is the first identified tight junction component [28, 29]. The effects of the *P. lobata* extract on the expression of tight junction proteins and membrane permeability have not been evaluated thus far. In the present study, we reported for the first time that the expression of tight junction proteins decreased in H_2_O_2_-treated RPE cells and that the *P. lobata* extract inhibited the disruption of ZO-1 expression, as observed by western blotting and immunohistochemistry (Figures 4 and 5). Tight junction disruption is related to the p38 MAPK signaling pathway [30, 31]. Inhibition or knockdown of JNK attenuates tight junction disruption [32]. The *P. lobata* extract inhibited p38 MAPK and JNK phosphorylation related to H_2_O_2_-induced tight junction disruption and barrier dysfunction. Pretreatment with *P. lobabta* extract showed preventive effects against oxidation, cell death, and tight junction disruption in H_2_O_2_-treated RPE cells. However, pretreatment with the individual compounds from the *P. lobata* extract did not show high efficiency compared to that obtained with *P. lobata* extract pretreatment. Therefore, these findings indicate that the *P. lobata* extract displays a synergistic effect of the three compounds. *P. lobata* extract is a crude herbal extract and is difficult to apply direct to the eyes now. However, following preclinical studies in the animal model for retinopathy and AMD and clinical studies, we can consider its clinical use *via* oral administration or eye drops. A variety of herb eye drops for allergies or eye health are currently on the market [33]. Future preclinical and clinical studies are required to further establish the effects of *P. lobata* extract.

## 5. Conclusions

The present study showed that the preventive effect of the *P. lobata* extract involved the inhibition of ROS generation and cell death in RPE cells. Furthermore, the *P. lobata* extract prevented tight junction disruption via the p38 MAPK and JNK signaling pathways. Together, these results strongly suggest that the *P. lobata* extract could be a potential alternative for preventing the development of oxidative stress-related ocular disorders such as AMD.

## Figures and Tables

**Table 1:** Calibration data of puerarin, daidzein, and daidzin.

Compound	Linear range (*μ*g/mL)	Regression equation^a^	Correlation coefficient (*R*^2^)
Puerarin	500–125	*y* = 24.354*x* − 155.57	0.9999
Daidzein	10–2.5	*y* = 31.088*x* − 2.6734	0.9999
Daidzin	100–25	*y* = 17.445*x* − 14.484	0.9999

^a^ *y*: peak area (mAU) of the component; *x*: concentration (*μ*g/mL) of the component.

**Table 2:** Contents of puerarin, daidzein, and daidzin in the *P. lobata* extract.

Compounds	Content (mean ± SD, *n* = 3)	
	*μ*g/mg	(%)
Puerarin	188.90 ± 2.22	18.9
Daidzein	2.73 ± 0.03	0.3
Daidzin	32.54 ± 0.45	3.3

## Data Availability

The data used to support the findings of this study are available from the corresponding author upon request.
